# Frequency and coinfection between genotypes of human papillomavirus in a population of asymptomatic women in northern Peru

**DOI:** 10.1186/s13104-018-3644-7

**Published:** 2018-07-31

**Authors:** Luis Ponce-Benavente, Patricia Rejas-Pinelo, Miguel Angel Aguilar-luis, Carlos Palomares-Reyes, Lorena Becerra-Goicochea, Luis Pinillos-Vilca, Wilmer Silva-Caso, Luis E. Costa, Pablo Weilg, Juan Alvitrez-Arana, Jorge Bazán-Mayra, Juana del Valle-Mendoza

**Affiliations:** 1 School of Medicine, Research and Innovation Centre of the Faculty of Health Sciences, Universidad Peruana de Ciencias Aplicadas, Lima, Peru; 2 Laboratorio de Biología Molecular, Instituto de Investigación Nutricional, Lima, Peru; 3 Instituto de Investigación de Enfermedades Infecciosas, Lima, Peru; 4 Hospital Regional Docente de Cajamarca, Cajamarca, Peru; 5 Universidad Viña del Mar, Viña del Mar, Chile; 6 Dirección Regional de Salud de Cajamarca (DIRESA), Cajamarca, Peru

**Keywords:** HPV, Cervical cancer, PCR, Peru

## Abstract

**Objective:**

Describe the prevalence of HPV genotypes via PCR and DNA sequencing in 397 women who attended to the gynecological outpatient center in the Hospital Regional Docente de Cajamarca from March to September 2017.

**Results:**

A positive PCR result for HPV was observed in 121 cervical samples. A high-risk genotype was found in 63.6% (77/121) of patients, a probably oncogenic type in 23.1% (28/121) and a low-risk type in 7.4%. Among the high-risk genotypes, HPV-31 was the most common one present in 20% (21/77), followed by HPV-16 in 11.4% (12/77). Coinfections between two or more genotypes were observed in 12 cases.

**Electronic supplementary material:**

The online version of this article (10.1186/s13104-018-3644-7) contains supplementary material, which is available to authorized users.

## Introduction

Cervical cancer is the second leading cause of female cancer mortality in Peru accounting for more than 1700 deaths annually [1]. Human papillomavirus (HPV) is the main responsible for this cancer and one of the most common sexually transmitted infection in Peru [2, 3]. HPV is a DNA virus from the papillomavirus family with over 170 types completely sequenced. Although the association between these genotypes and their risk of malignancy varies, its presence in 99.7% of cervical cancers has been recognized as core to the development of this neoplasia [4, 5].

HPV genotypes identified in women with cervical cancer are traditionally classified based on their associated risk of malignancy [5, 6]. Thus, 13 genotypes have been classified as “high risk” as they are detected in more than 90% of women with cervical cancer, with HPV 16 accounting for approximately 50% of cases, followed by HPV 18 in 20% [6–9]. Furthermore, in 2009 the International Agency for Research on Cancer (IARC) further classified other 12 genotypes as “probably oncogenic” and 2 as “low risk” based on their limited evidence in humans for cervical cancer [9].

After over 30 years of screening services and with cervical cancer declared a national priority in 2000, HPV infections remains a major concern in Peru especially affecting underserved areas where surveillance is limited [3, 10–12]. The main objective of this study was to describe the prevalence of the HPV oncogenic genotypes among women from Cajamarca, Peru as well as to provide preliminary reports of their potential risk factors.

## Main text

### Methods

#### Patients and study design

A consecutive cross-sectional study was conducted in the *Hospital Regional Docente de Cajamarca*, Peru. Women attending the gynecological outpatient center who had a history of at least 1 sexual encounter were studied from March to September 2017. Patients were excluded if there was evidence of pregnancy, severe gynecological bleeding, hysterectomy or previous history of HPV-related disease including cancer, warts and other cutaneous manifestations.

This study has been approved by two independent Ethics Committees: *Hospital Regional Docente de Cajamarca* and *Universidad Peruana de Ciencias Aplicadas*. Once the outpatient consultation ended, the attending physicians informed the patients about the study purpose and asked for written consent before enrollment. After a standardized questionnaire was completed by the physician a cervical sample was collected for further analysis. The questionnaire includes the following data: age, marital status, age of first sexual intercourse, number of sexual partners, sexual partners in the last 6 months, extramarital sexual relations, history of sexually transmitted infection (STI), abortions, Number of births, use of condoms, last pap, personal and family history of cervical cancer, use of sex toys, history of sexual abuse and date of the Last Papanicolaou test.

#### Sample collection and preservation

Cervical cell samples from the ectocervix and endocervix were collected from each woman using a cytobrush for preservation in a tube containing phosphate buffered saline (pH 8.6). The samples were then stored at − 4 °C and sent to the Molecular Biology Laboratory at *Universidad Peruana de Ciencias Aplicadas*. Once samples arrived at the laboratory, the cytobrushes were discarded and the tubes were vortexed and centrifuged to pellet the cells, which were resuspended in 1 mL of phosphate buffered saline. Three aliquots of each fresh specimen were stored at − 20 °C until testing.

#### HPV DNA extraction, amplification and genotype sequencing

Viral genomic DNA was extracted from a total volume of 200 μL of the sample by the guanidinium thiocyanate extraction method [13] and the purified material was re-suspended in a final volume of 30 μL deionized water. Samples were electrophoresed on a 1% agarose gel to check the quality of the DNA.

Human papillomavirus amplification was done using the primers and conditions described by Lurchachaiwong et al. [14]. PCR products were analyzed on 2% agarose gel stained with.

ethidium bromide and bands were detected by UV transillumination (Kodack Logic 1500, USA). Positive specimens were confirmed by direct sequencing serving as the gold standard (Macrogen-Korea).

The HPV genotypes were categorized into three groups: high, probably oncogenic and low risk based on the IARC classification [9] (Additional file 1: Table S1).

#### Statistical analysis

Quantitative variables were described as frequencies and percentages for each group using the GraphPad Prism3 statistical (Graph Pad Sofware Inc., San Diego, USA).

### Results

A total of 397 women were studied from March to September 2017. Most patients were between 36 and 45 years old (35%) followed closely by the age group 26–35 years old (30.2%); 51 patients were under 25 years old (12.8%) with only two patients under 18 years old (Table 1).

**Table 1:** Human papillomavirus infection in women from Cajamarca, Peru

Age (years)	Total cases n = 397 (%)	Positives cases of HPV n = 121 (%)	HPV genotypes detected			
			High risk n = 77 (%)	Probably oncogenic n = 28 (%)	Low risk n = 9 (%)	Other types of HPV^a^ n = 24 (%)
18–25	51 (12.8)	22 (18.2)	16 (20.8)	4 (14.3)	2 (22.2)	3 (12.5)
26–35	120 (30.2)	43 (35.5)	30 (38.9)	9 (32.1)	3 (33.3)	7 (29.2)
36–45	139 (35)	41 (33.9)	23 (29.9)	12 (42.9)	4 (44.5)	8 (33.3)
≥ 46	87 (21.9)	15 (12.4)	8 (10.4)	3 (10.7)	0 (0.0)	6 (25.0)
Total	397 (100.0)	121 (100.0)	77 (100.0)	28 (100)	9 (100.0)	24 (100.0)

^a^Other HPV types: 9, 40, 42, 43, 44, 74, 90, 91, 96

Human papillomavirus DNA was amplified in 121 of our patient’s samples and all of them were successfully sequenced for genotype identification. A high-risk genotype was found in 63.6% (77/121) of patients, a probably oncogenic type in 23.1% (28/121) and a low-risk type in 7.4%; other genotypes were detected in 19.83%. All sequenced genotypes are shown in Table 2.

**Table 2:** Prevalence of HPV genotypes detected in women from Cajamarca, Peru

Genotypes detected	Frequency	Percentage
High risk (included probably oncogenic)	n = 105	(%)
HPV16	12	11.4
HPV18	1	1
HPV31	21	20
HPV33	3	2.9
HPV35	8	7.6
HPV39	2	1.9
HPV45	4	3.8
HPV51	4	3.8
HPV52	9	8.6
HPV56	5	4.8
HPV58	2	1.9
HPV59	1	1
HPV68	5	4.8
HPV26	1	1
HPV34	7	6.7
HPV53	2	1.9
HPV66	2	1.9
HPV69	5	4.8
HPV70	9	8.6
HPV73	2	1.9

Low risk	n = 9	
HPV6	8	88.9
HPV11	1	11.1

Other HPV types	n = 24	
HPV9	1	4.2
HPV40	2	8.3
HPV42	2	8.3
HPV43	2	8.3
HPV44	1	4.2
HPV74	2	8.3
HPV90	9	37.5
HPV91	4	16.7
HPV96	1	4.2

Coinfections between two or more genotypes were observed in 12 cases. The most common coinfections were between HPV types 39–45–68, 40–43–91 and 31–91 each of them in two cases (Additional file 1: Table S2).

Demographic and other potential risk factors for HPV infections were also registered from each patient. Most women were married/cohabiting, had one sexual partner, maintaining sexual relationships in the last 6 months. However, only 35.5% of them uses condoms and the use of sex toys was uncommon. Extramarital affairs were observed in 3.3% of patients and there were 31 cases with a history of sexual abuse. Most patients were multiparous with three or more births (36.8%) (Table 3).

**Table 3:** Demographics and characteristics among women with HPV

Characteristics	Total cases n = 397 (%)	Positives cases of HPV n = 121 (%)	HPV genotypes detected			
			High risk n = 77 (%)	Probably oncogenic n = 28 (%)	Low risk n = 9 (%)	Other types of HPV n = 24 (%)
Marital status						
Married/cohabiting	281 (70.8)	76 (62.8)	47 (61.0)	21 (75.0)	4 (44.4)	13 (54.2)
Single/separated/divorced/widowed	116 (29.2)	45 (37.2)	30 (39.0)	7 (25.0)	5 (55.6)	11 (45.8)
Lifetime number of sexual partners						
1	213 (53.7)	51 (42.1)	28 (36.4)	12 (42.9)	3 (33.3)	16 (66.7)
2	114 (28.7)	39 (32.2)	23 (29.9)	13 (46.4)	2 (22.2)	6 (25.0)
≥ 3	69 (17.4)	31 (25.6)	26 (33.8)	3 (10.7)	4 (44.4)	2 (8.3)
Number of sexual partners in the last 6 months						
0	55 (13.9)	20 (16.5)	12 (15.6)	4 (14.3)	3 (33.3)	3 (12.5)
1	336 (84.6)	97 (80.2)	62 (80.5)	23 (82.1)	6 (66.7)	21 (87.5)
≥ 2	6 (1.5)	4 (3.3)	3 (3.9)	1 (3.6)		
Use of condom						
Yes	141 (35.5)	53 (43.8)	35 (45.5)	12 (42.9)	2 (22.2)	10 (41.7)
No	256 (64.5)	68 (56.2)	42 (54.5)	16 (57.1)	7 (77.8)	14 (58.3)
Use of sex toys						
Yes	8 (2.0)	4 (3.3)	2 (2.6)	1 (3.6)	–	3 (12.5)
No	389 (98.0)	117 (96.7)	75 (97.4)	27 (96.4)	9 (100.0)	21 (87.5)
Extramarital affairs						
Yes	13 (3.3)	6 (5.0)	7 (9.1)	1 (3.6)	–	–
No	384 (96.7)	115 (95.0)	70 (90.9)	27 (96.4)	9 (100.0)	24 (100.0)
Victim of sexual abuse						
Yes	31 (7.8)	12 (9.9)	8 (10.4)	4 (14.3)	–	1 (4.2)
No	366 (92.2)	109 (90.1)	69 (89.6)	24 (85.7)	9 (100.0)	23 (95.8)
Date of the last papanicolaou test						
Never	69 (17.4)	24 (19.8)	19 (24.7)	6 (21.4)	–	7 (29.2)
≤ 1 year	199 (50.1)	59 (48.8)	40 (51.9)	12 (42.9)	5 (55.6)	9 (37.5)
≥ 2 years	129 (32.5)	38 (31.4)	18 (23.4)	10 (35.7)	4 (44.4)	8 (33.3)
Number of births						
0	58 (14.6)	28 (23.1)	20 (26)	3 (10.7)	3 (33.3)	6 (25.0)
1	90 (22.7)	34 (28.1)	21 (27.3)	9 (32.1)	1 (11.1)	11 (45.8)
2	103 (25.9)	29 (24)	16 (20.8)	8 (28.6)	2 (22.2)	4 (16.7)
≥ 3	146 (36.8)	30 (24.8)	20 (26)	8 (28.5)	3 (33.3)	3 (12.5)
Number of abortions						
0	281 (70.8)	89 (73.6)	59 (76.6)	18 (64.3)	7 (77.8)	20 (83.4)
1	86 (21.7)	21 (17.4)	12 (15.6)	6 (21.4)	2 (22.2)	2 (8.3)
2	23 (5.8)	8 (6.6)	4 (5.2)	3 (10.7)	–	2 (8.3)
3	7 (1.8)	3 (2.5)	2 (2.6)	1 (3.6)	–	–

*HPV* human papillomavirus

Among women who were infected by a high-risk HPV genotype, similar characteristics were observed with certain exceptions. In this group, it was slightly more common to observe 2-lifetime sexual partners and the majority were sexually active (82.1%). Although uncommon, 53.8% of extramarital affairs were observed in this group. Additionally, 19 of these women have never had a Pap smear test for cervical cancer screening (Table 3).

### Discussion

Cervical cancer is the most common female cancer in Peruvian women between 15 and 44 years-old, with about 4700 new cases diagnosed annually and an estimated mortality of 24.6 per 100,000 women [3, 10]. There is clear evidence that HPV is the main responsible for cervical cancer and prevalence studies of the oncogenic genotypes are encourage especially in low-income communities were surveillance reports are still limited [3, 10–12, 15].

In our study population of 397 women, a total of 121 samples were positive for HPV via PCR, with a high-risk genotype present in 77 samples and a probably oncogenic genotype in 28. In the high-risk group, the most common genotypes were HPV-31 (20%) followed by HPV-16 (11.4%). These results differ from a previous investigation our research team conducted in Cajamarca between 2010 and 2012, in which the HPV-16 was the most common genotype in 38.5%, followed by the HPV-39 in 9.6% [15].

Another investigation in 465 women, without the diagnosis of cervical cancer, from Lima, Peru reported the genotypes HPV-16 (23.8%) and HPV-6 (11.9%) [16] as the most common ones. Furthermore, a study conducted in an Amazonian region of Peru showed a higher frequency of high-risk HPV among females from an urban population in Iquitos compared to a native Amazonian population. However, the genotypes distribution in both population were different. In the urban population, HPV-16 was the most common type in 58.5% followed by HPV-18 and HPV-31. On the contrary, in the Amazonian native community, HPV-16 was uncommon, as other unique HPV types were more frequently observed in these patients such as HPV-39, HPV-71, and HPV-96 [17]. Thus, HPV genotypes distribution can widely vary between two different close regions from the same country and changes in genotype prevalence are expected, with studies reporting an HPV-16 prevalence significant decrease in the last years after the introduction of the vaccine [18–20].

Multiple genotypes can be detected in patients with cervical cancer suggesting that coinfections might be a potential risk factor for carcinogenesis. A recently published study from 2017, reported a 4.1-fold higher risk of developing invasive cervical carcinoma in subjects infected with any HPV genotype, excluding HPV-16, in association with HPV-18 [21]. Thus, the risk of cervical cancer is genotype specific, but it might be increased with certain genotype interactions. In our study, only one case of HPV-16 coinfection was observed, but coinfections with a high-risk type occurred in 5 samples, including a triple coinfection between HPV-39, HPV-45, and HPV-68.

Genital HPV infections are spread by unprotected penetrative intercourse or close skin-to-skin physician contact [22, 23]. In our population, most patients did not use condoms (64.5%), and this increased risk practice was observed in all our HPV-positive patients (56.2%), high-risk HPV positive (54.5%) and probably oncogenic HPV positive women (57.1%). Fomite contact or vaginally inserted sex toys can potentially spread HPV as the virus can be detected up to 24 h after standard cleaning, but the evidence is not definitive. The use of sexual toys was very uncommon in our patients (2%), with a positive HPV sample in 4 of the 8 cases.

The risk of HPV infection in women is directly related to the number of male sex partners. Furthermore, as with other sexually transmitted diseases, sex with a new partner is a stronger risk factor than sex with long-term partners [24–28]. In our study, females with 3 or more partners represented 25.6% of HPV-positive patients. Additionally, 4 cases of HPV were observed in females who had 2 or more partners in the last 6 months; although most of our patients with HPV (80.2%) were sexually active with 1 partner in the last 6 months. Similar results were observed in our previous investigation in which the groups of women with a history of 2 sexual partners and the group with 3 or more partners represented 25% and 17.3% of the total HPV positive cases [15].

In 2011, Almonte et al. conducted a research in women attending cervical cancer screening in the Peruvian Amazon. In this study, early age at first sexual intercourse and more than 5 sexual partners were risk factors for having HPV infection. More interestingly, high parity, no schooling and the lack of a good-quality screening with an adequate follow-up were the main risk factors for high-grade cervical disease [29]. Even though, assessing a risk for cervical disease was not part of our objectives, we observed that 36.8% of our population had 3 or more births and a similar tendency was reported among HPV positive women.

Human papillomavirus DNA testing is increasingly being used as it improves the sensitivity for detection of cervical cancer precursors when used in combination with cervical cytology. However, using molecular testing can also decrease specificity resulting in potential unnecessary referrals for colposcopy [30]. Additionally, HPV PCR is not recommended in women under 30 years old since the HPV may clear up spontaneously in younger women [31, 32]. Thus, the Papanicolaou test is recommended as the first screening method with a co-testing HPV PCR only in women ≥ 30 years old. In our population, 24.7% (19/77) of our HPV high-risk group never had a pap smear; however, most of these women (73.7%) were under 30 years old.

### Conclusion

Despite national efforts for cervical cancer screening and prevention, HPV infections resulting in invasive cervical cancer remains a major health issue in Peru. Additionally, in underserved areas such as Cajamarca HPV genotype surveillance is limited. Contrary to previous reports in which HPV-16 was predominant, an increase of HPV-31 have been observed which is now the most common high-risk genotype in our study population.

## Limitations

The present study had one important limitations. We designed the study for the detection and genotypification of HPV, thus our results are only for prevalence reporting and the implications of these genotypes or the potential risk for cancer in our patients is unknown. Second, due financial limitations and our study design it was impossible to compare the isolated genotypes with pap smear results as they were not available in most of our patients.

## Additional file

**Additional file 1: Table S1.** Human papillomavirus types and oncogenic potential. **Table S2.** Demographics and characteristics among women with HPV.

## Abbreviations

PCR: polymerase chain reaction
DNA: deoxibonucleic acid
bp: base pairs
HPV: human papillomavirus
PBS: phosphate buffered saline
