# Supplementary material for: Frequency and coinfection between genotypes of human papillomavirus in a population of asymptomatic women in northern Peru
Source: BMC Res Notes. 2018 Jul 31;11:530. doi: 10.1186/s13104-018-3644-7 (PMC6069864; doi:10.1186/s13104-018-3644-7)
Supplement: Supplementary file 1 — Additional file 1: Table S1. Human papillomavirus types and oncogenic potential. Table S2. Demographics and characteristics among women with HPV. [file 13104_2018_3644_MOESM1_ESM.docx]

**Table S1.** Human papillomavirus types and oncogenic potential.

| **IARC Classification** | **HPV types** |
| --- | --- |
| **High risk (group 1/2A)^a^** | 16, 18, 31, 33, 35, 39, 45, 51, 52, 56, 58, 59, 68 |
| **Probably oncogenic (group 2B)^b^** | 26, 30, 34, 53, 66, 67, 69, 70, 73, 82, 85, 97 |
| **Low risk** | 6, 11 |

HPV = human papillomavirus.

a. Sufficient evidence for cervical cancer

b. Limited evidence for cervical cancer and classified by phylogenetic analogy to HPV types 1/2A

**Table S2.** Coinfections between different HPV genotypes

| **Patients with coinfections**  **of HPV types** | **n = 13** | **(%)** |
| --- | --- | --- |
| HPV39, HPV45, HPV68 | 2 | 15.4 |
| HPV40, HPV43, HPV91 | 2 | 15.4 |
| HPV31, HPV90 | 2 | 15.4 |
| HPV31, HPV53 | 1 | 7.7 |
| HPV26, HPV69 | 1 | 7.7 |
| HPV16, HPV52 | 1 | 7.7 |
| HPV56, HPV66 | 1 | 7.7 |
| HPV33, HPV52 | 1 | 7.7 |
| HPV9, HPV16 | 1 | 7.7 |
| HPV66, HPV90 | 1 | 7.7 |

Red = High-risk genotype, Blue = Probably oncogenic, Black = Others
